# Quantification of Gram-positive bacteria: adaptation and evaluation of a preparation strategy using high amounts of clinical tissue

**DOI:** 10.1186/1746-6148-10-53

**Published:** 2014-03-03

**Authors:** Evelyne Mann, Katharina Pommer, Patrick Mester, Martin Wagner, Peter Rossmanith

**Affiliations:** 1 Department of Veterinary Public Health and Food Science, Institute of Milk Hygiene, Milk Technology and Food Science, University of Veterinary Medicine Vienna, Veterinaerplatz 1, Vienna 1210, Austria; 2 Department of Veterinary Public Health and Food Science, Christian Doppler Laboratory for Molecular Food Analytics, University of Veterinary Medicine, Veterinaerplatz 1, Vienna 1210, Austria; 3 Department of Veterinary Public Health and Food Science, Christian Doppler Laboratory for Monitoring of Microbial Contaminants, University of Veterinary Medicine, Veterinaerplatz 1, Vienna 1210, Austria

**Keywords:** Matrix-Lysis, Pathogen detection, Pre-PCR, Tissue preparation, Bacteria quantification

## Abstract

**Background:**

A preparation method for quantification of bacteria in tissues is obligatory to reduce tissue mass, concentrate the target, purify, remove inhibitory substances and to achieve constant target recovery rates. No preparation method has been available until now for a high mass of tissue applicable for routine use and analytical veterinary diagnostics.

**Results:**

This study describes an easy-to-use tissue preparation protocol to quantify Gram-positive bacteria from a large volume of tissue matrix. A previously published sample preparation method (Matrix-Lysis) from food science was successfully adapted for clinical use on tissues from pigs, including cerebrum, spinal cord, lung, liver, ileum, colon, caecum, kidney and muscle tissue. This tissue preparation method now permits quantification of pathogens from 5 g of organic matrix, which is a 20–200 fold increase by weight compared to other methods. It is based on solubilization of the sample matrix with either a chaotrope plus detergent or divalent salts as solubilization agents. The method was designed as a modular system, offering the possibility to change lysis buffers, according to tissue solubilization characteristics and the intended detection method (molecular or culture). Using *Listeria monocytogenes* as model organism, viable cell quantification or DNA extraction and quantitative real-time PCR were performed after Matrix-Lysis to determine recovery rates and detection limit (LOD). The adapted Matrix-Lysis protocol resulted in high recovery rates (mean value: 76% ± 39%) for all tested organs, except kidney, and recovery was constant over 5 log scales for all tested buffer systems. The LOD for Matrix-Lysis with subsequent plate count method (PCM) was as low as 1 CFU/5 g, while for qPCR based detection the LOD was 10^2^ bacterial cell equivalents (BCE)/5 g for two buffer systems.

**Conclusions:**

This tissue preparation is inexpensive and can be easily used for routine and analytical veterinary diagnostics. Inoculation studies or hazard assessments can profit from this tissue preparation method and it is anticipated that this study will be a valuable source for further research on tissue preparation strategies.

## Background

Molecular tools for detecting, analyzing and quantifying specific microorganisms are well established in modern analytical diagnostics. The rising demand for fast quantification tools for pathogens has led to a tremendous increase in methodological approaches focusing on molecular biological techniques, such as quantitative real-time PCR (qPCR) [1-3]. Real-time PCR is a very efficient and accurate quantification technique with a potential limit of detection (LOD) of three target copies per PCR reaction, assuming Poisson distribution and a confidence level of 95% [4].

PCR assays have been published for almost all known bacterial pathogens, targeting resistance or virulence genes or conserved regions [5,6]. However, prior methods that have to be used before qPCR assays, such as tissue preparation or DNA extraction, are disregarded in most cases. In analytical diagnostics, qPCR is mostly applied after direct DNA extraction using a small amount of tissue matrix (25–250 mg). This is performed without any appropriate tissue preparation strategy, where target cells are extracted, concentrated and purified from the surrounding matrix [7-10]. This approach can result in false quantification of bacterial targets and, even more importantly, false negatives in qPCR [2,11]. The main reasons for these complications are concentration and irregular distribution of target cells in tissues: when skipping tissue preparation and directly processing a small quantity via DNA extraction, pathogens that are not uniformly distributed in the tissue might be over- or underestimated. This can also be fostered by heterogeneity of the tissue matrix, by pathological tissue alterations or by a low number of target microorganisms. Therefore, a representative quantity of tissue and subsequent tissue preparation to concentrate target cells are essential for precise quantification.

Besides qPCR, other methods can benefit from the abilities of a tissue preparation method to reduce the tissue matrix and concentrate target bacteria. For example, plating microorganisms is a common approach to qualitative pathogen detection, but where enrichment steps are necessary prior to detection, quantification becomes impossible. A high background flora on plates might strongly influence the growth of target organisms. This, as well as the methodological problem of low-occurrence of pathogens, is frequently discussed in the literature [12-14]. A direct plating approach, to quantify low numbers of target cells, has not been used in clinical diagnostics until now. However, this is possible using an adequate tissue preparation method to concentrate target cells prior to plating.

In general, pathogen quantification is always a multistep process, which is also referred to as an analytical chain. This chain can be subdivided into links comprising “sampling, tissue preparation, and plating” or “sampling, tissue preparation, DNA extraction/isolation, and target amplification/detection” [15]. Each individual link within such a chain is equally important and this necessitates that they should be individually controlled. A failure at any one link in the chain falsifies the final result. Another consequence of such an analytical chain is that every upstream method constitutes a bottleneck for the subsequent method. Targets lost early cannot be “recovered” later. It therefore becomes clear that the initial links within an analytical chain, in this case pre-treatment and tissue preparation, are key in analyses and that they are as important as later DNA-extraction or target amplification.

Implementation of a cost-effective, robust and easy-to-use tissue preparation method prior to pathogen detection remains challenging in clinical applications, and is still problematic in nucleic acid testing [16]. Until now no reliable tissue preparation method for routine applications and analytical veterinary diagnostics has been available. Possible solutions to these difficulties could come from other disciplines. A scientific field that faces the same challenges as clinical diagnostics, namely the need to detect pathogens in a large quantity of a complex matrix, qualitatively, and especially quantitatively, is food safety.

In food microbiology, requirements for a food sample preparation method are now well defined and can be specified as follows: i) reduction of matrix size for downstream steps without loss of target bacteria, ii) concentration of the target, iii) purification and removal of inhibitory substances, and iv) a low detection limit and constant recovery rate of the target [17]. Many sample preparation techniques have been described in the literature that aim to concentrate target cells and purify them from extraneous matrices: immunological methods based on magnetic bead separation [18] or high gradient separation [19], physical methods based on filtration, flotation or gradient centrifugation [20,21], adsorptive methods using activated carbon for separation of target cells [22] and methods based on electrophoresis or enzymatic digestion [22,23]. Due to limitations of these methods, including high costs, low throughput, complex procedures or the need for minimum, non-representative tissue quantities [24], these methods have largely been abandoned in analytical veterinary diagnostics. It is unlikely that any one of these aforementioned strategies can cover all requirements for organs, including their pathological variants and species-specific differences.

However, a promising sample preparation method that can overcome these problems is Matrix-Lysis, which was initially developed in food science for application with dairy and meat products [25]. The Matrix-Lysis protocol includes solubilization of the food matrix, concentration and purification of contaminant bacteria through washing and centrifugation steps. The method is economical, rapid and the LOD for food matrices has been described to be lower than 10 CFU/g [25-27]. In particular, Matrix-Lysis is a modular system with interchangeable lysis buffers. The lysis buffer is responsible for adequate solubilization of the matrix. If the matrix is well solubilized, the size of the remaining pellet (consisting of both bacteria and some tissue residues) is small, as most of the tissue is dissolved. This is important as only small pellets (<350 mg) can be processed further in a DNA extraction system; otherwise the background DNA is too high or the column of the DNA extraction system becomes obstructed. Interchangeability of the lysis buffers permits adaptations, alterations and structural changes, depending upon the matrix components, which could be highly advantageous for clinical applications.

The aim of this study was therefore to apply and adapt the Matrix-Lysis food sample preparation method to different healthy organs and to introduce it as a tissue preparation method for routine use and analytical veterinary diagnostics. The end objective was to achieve a detection limit of <100 BCE (bacterial cell equivalents)/g, which is claimed to be required urgently in diagnostics [16]. A method such as this is anticipated to improve quantification processes in veterinary clinical research, microbiology and post-mortem diagnostics. The study organs were taken from healthy slaughtered pig carcasses and acted as models for tissues originating from a variety of mammalian species. Furthermore, detection limits for the analytical chains “Matrix-Lysis, DNA-extraction and qPCR”, and “Matrix-Lysis and subsequent plate count method (PCM)” were defined. For spiking experiments, *Listeria (L.) monocytogenes* was used as a model Gram-positive organism.

## Results

### Solubilization of clinical tissues and adaptation of the Matrix-Lysis protocol

The Matrix-Lysis protocol was applied as published [26], for testing the solubilization capability of two different lysis buffers (8 M urea + 1% SDS; 2 M MgCl_2_ with optional 1% Lutensol AO-07) on clinical tissues. The solubilization capability was considered sufficient if the remaining pellet after Matrix-Lysis was small enough for downstream analysis. The threshold for the pellet size was set at <350 mg. This corresponded to a pellet mass of 7% compared to the mass utilized (5 g). A remaining pellet size >7% was considered insufficiently solubilized. Six out of nine clinical tissues were solubilized sufficiently using the 8 M urea + 1% SDS lysis buffer. Insufficient lysis was achieved with lung, ileum and caecum. Only three sufficient tissue lysates were obtained using the 2 M MgCl_2_ lysis buffer. Liver, colon, kidney, lung, ileum and muscle tissue produced pellets more than 7% volume. The resultant pellet size from five tissues was considered sufficient with the 1% Lutensol AO-07 added to the 2 M MgCl_2_ lysis buffer. Liver, lung, ileum and caecum could not be lysed sufficiently. Details about the remaining pellet sizes are listed in Table 1.

**Table 1:** Remaining pellet size (% wet weight) after performing Matrix-Lysis protocol with different lysis buffers and tissues

	**8 M urea + 1% SDS (%)**		**2 M MgCl**_**2**_ **+ 1% Lutensol (%)**		**2 M MgCl**_**2 **_**(%)**	
	**Mayrl et al. (2009) **[26]	**This study**	**Mayrl et al. (2009) **[26]	**This study**	**Mayrl et al. (2009) **[26]	**This study**
Cerebrum	1.1 ± 0.12	n.e.	2.9 ± 0.05	n.e.	1.4 ± 0.20	n.e.
Spinal cord	1.3 ± 0.11	n.e.	2.5 ± 0.17	n.e.	1.7 ± 0.25	n.e.
Liver	1.8 ± 0.31	n.e.	7.2 ± 0.24	4.3 ± 0.10	8.2 ± 0.21	3.9 ± 0.04
Colon	2.7 ± 0.16	n.e.	9.8 ± 0.03	2.2 ± 0.13	5.2 ± 0.80	n.e.
Kidney	1.0 ± 0.00	n.e.	7.2 ± 0.43	4.1 ± 0.03	3.4 ± 0.25	n.e.
Lung	13.2 ± 0.20	9.8 ± 0.12^1^	23 .0 ± 0.18	7.3 ± 0.17^1^	56.0 ± 0.35	6.6 ± 0.43
Ileum	9.8 ± 0.30	2.6 ± 0.11	47.0 ± 0.09	2.9 ± 0.05	52.0 ± 0.20	3.1 ± 0.05
Caecum	12.1 ± 0.15	3.8 ± 0.10	3.7 ± 0.04	n.e.	12.0 ± 0.24	4.3 ± 0.10
Muscle	1.7 ± 0.08	n.e.	26.7 ± 0.47	11 .0 ± 0.31^1^	5.9 ± 0.02	n.e.

^1^lysis efficiency was too low for sufficient solubilization; these pellets could not be used for further downstream analyses.

n.e. = not examined (the protocol of Mayrl et al. (2009) [26] revealed sufficient solubilization).

**Figure 1:**
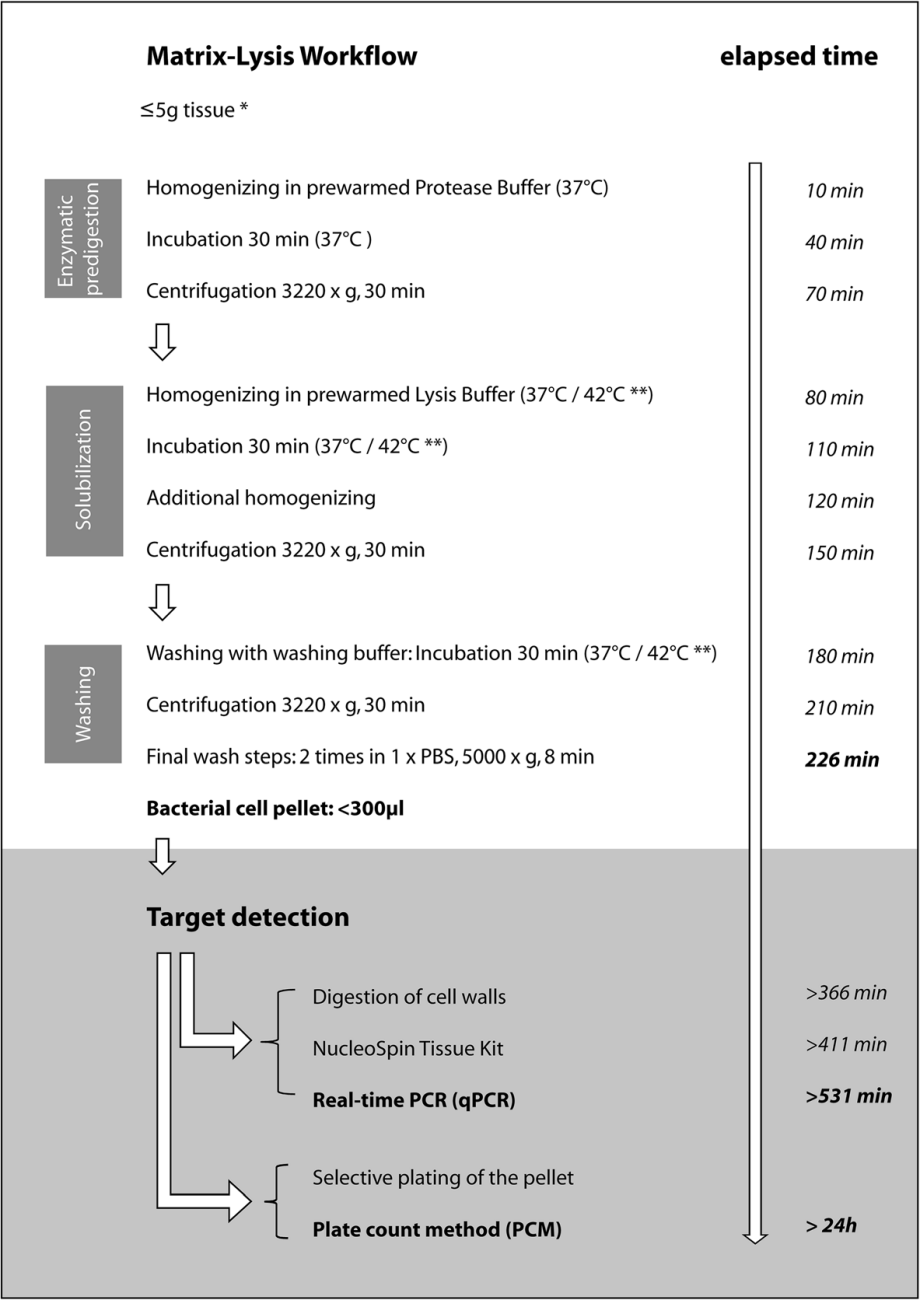
**Matrix-Lysis protocol adapted for clinical tissue.** This includes timeline and subsequent target detection paths. *The protocol is designed for a maximum of 5 g of tissue. The tissue must be cut into small pieces (Ø <4 mm^2^). **Homogenization of tissue in 8 M urea + 1% SDS takes place at an incubation temperature of 42°C; an incubation temperature of 37°C is recommended for 2 M MgCl_2_ (±1% Lutensol).

Matrix-Lysis was repeated making use of the adapted protocol (this study) to achieve more efficient tissue lysis. An additional homogenization step after incubation in lysis buffer was introduced and pre-warmed buffers (protease buffer, lysis buffer and washing buffer) further enhanced the solubilization process. The complete protocol established for clinical tissue is shown in Figure 1. All tissues that were insufficiently lysed were tested again with the established Matrix-Lysis protocol for clinical tissue. The 8 M urea + 1% SDS lysis buffer resulted in sufficient pellet sizes for all tissues, except lung. The 2 M MgCl_2_ lysis buffer was insufficient for lung and muscle tissues. However, all tissues were solubilized sufficiently using the 2 M MgCl_2_ + 1% Lutensol AO-07 lysis buffer (Table 1).

### Artificial contamination experiments

**Figure 2:**
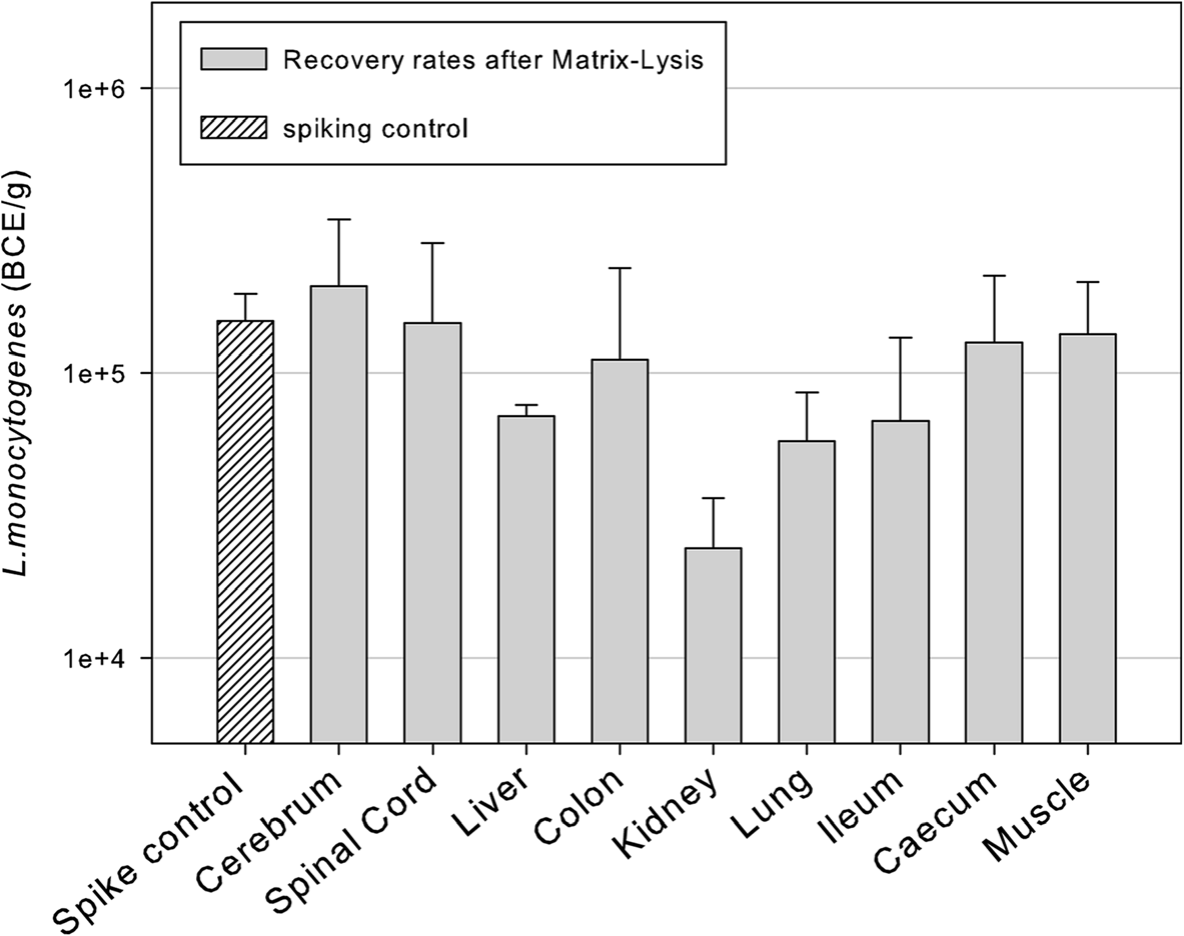
**Recovery of *L. monocytogenes *(BCE/g) after Matrix-Lysis from nine different clinical tissues.** The spike control represents the initial number of BCE/g that was used for spiking clinical tissues. The lysis buffer based on 2 M MgCl_2_ + 1% Lutensol was used for Matrix-Lysis. Bars indicate standard deviations.

Results of artificial contamination experiments for nine different tissues are presented in Figure 2. Matrix -Lysis, including the lysis buffer 2 M MgCl_2_ + 1% Lutensol, was used and subsequently DNA extraction and qPCR were performed for quantification. The tissues were spiked with approximately 10^5^ BCE/g. Overall recovery after Matrix-Lysis was within 1 log_10_ unit for all nine tissues. Unexpectedly, kidney tissue showed consistently lower recovery rates (15% ± 7.9%) compared with other organs. Mean recovery rate of target cells from organs, excluding kidney, was 76% ± 39%. To test the stability of recovery rates through dilution series, extended artificial contamination experiments over a five log_10_ dilution series were performed with 5 g of cerebral tissue. Lysis buffers used for Matrix-Lysis were 8 M urea + 1% SDS and 2 M MgCl_2_ + 1% Lutensol and qPCR was used for quantification. The amount of inocula ranged from 10^2^-10^6^ BCE/g tissue. With 8 M urea + 1% SDS buffer for Matrix-Lysis, mean recovery rate was 32.5% (*R*^*2*^ *=* 0.99). When the buffer was based on 2 M MgCl_2_ + 1% Lutensol, mean recovery was 104.3% (*R*^*2*^ *=* 0.99).

The PCM method (ALOA agar) was used for quantification of the 2 M MgCl_2_ lysis buffer-treated samples, since 2 M MgCl_2_ is known to preserve the viability of bacteria and this makes a plating approach possible. The amount of inocula ranged from 10^1^- 10^5^ CFU/g. The mean recovery rate for *L. monocytogenes* using Matrix-Lysis, with a lysis buffer based on 2 M MgCl_2_, was 96.1% (*R*^*2*^ *=* 0.99) after PCM.

### LOD for PCM

The major advantage of Matrix-Lysis tissue preparation lies in its efficiency and robustness to recover and concentrate as few as one bacterial cell from a large sample mass (5 g) into a pellet small enough for subsequent molecular and microbiological detection methods. To determine if tissue preparation with Matrix-Lysis is as efficient at low bacteria numbers as it is with higher numbers, tests were performed to determine both the LOD and reproducibility of the Matrix-Lysis tissue preparation method. This was performed by testing triplicates of low bacteria counts (between 0.5 and 50 CFU/5 g tissue; Table 2). Tests were performed using PCM as the detection method because only a fraction (in this study 1/20) of the total sample is used for the actual detection reaction with a qPCR detection system after DNA-extraction; inevitably resulting in a higher LOD [28].

**Table 2:** LOD determination of Matrix-Lysis tissue preparation using plate count method (PCM) for enumeration from 5 g of cerebral tissue

**CFU**^**1 **^**control**^**2**^	**CFU per plate after ML**^**3**^	**Sum of CFU after ML**
51 ± 3	21, 9, 11, 5, 11	57
4.5 ± 0.5	2, 0, 1, 1, 0	4
0.5 ± 0.5	1, 0, 0, 0, 0	1
18 ± 1	2, 10, 5, 10, 4	31
0.5 ± 0.5	0, 1, 0, 0, 0	1
0 ± 0	0, 0, 0, 0, 0	0
20 ± 5	4, 5, 7, 4, 1	21
1 ± 0	0, 3, 0, 0, 0	3
0 ± 0	0, 0, 0, 0, 0	0

^1^CFU: Colony forming units.

^2^PCM was used prior to Matrix-Lysis on TSA + Y for the spiking control.

^3^Recovery rates after Matrix-Lysis were determined by subdividing the remaining pellet into five equal parts and plating them individually onto ALOA agar.

Positive and negative controls performed as expected. The average number of CFU incorporated into each 5 g sample was obtained on TSA + Y agar. The number of CFU after Matrix-Lysis was determined by subdividing the remaining pellet into five equal parts and plating them individually on ALOA selective media. The LOD for *L. monocytogenes* from 5 g of cerebrum tissue with Matrix-Lysis + PCM was determined to be as low as one single cell and the efficiency of Matrix-Lysis tissue preparation at low levels was equal to that determined at higher bacterial levels (Table 2).

## Discussion

Detection, and especially quantification, of pathogens present in very low numbers in a large clinical sample requires technological solutions adapted to the respective tissue matrix [24]. It is essential for any molecular diagnostic to realize that the detection reaction is indeed only the last in a series of sequentially applied methods within an analytical chain consisting of sample preparation, target extraction, purification and, finally, detection. What concludes from recognition of the analytical chain is that a reliable and controllable method for each of these steps has to be chosen and examined, because if any of those methods fails, a false negative result is inevitable.

Although it is a definitive bottleneck for the whole analytical chain, sample preparation has not received as much attention in the past as it deserves. However, this is expected to change. Current efforts in sample preparation [25] and molecular enrichment [28] have demonstrated simple solutions for concentrating and recovering microorganisms. In this study we present Matrix-Lysis, which breaks up cell membranes and solubilizes the tissue matrix, thereby additionally permitting detection of intracellular bacteria. This is an important issue in clinical diagnostics, whereby a possible dissemination of pathogenic bacteria can be monitored.

In this study, for every tissue tested, more than one effective lysis strategy could be found. Lung tissue was an exception; only the 2 M MgCl_2_ + 1% Lutensol lysis buffer permitted an adequate resultant pellet size for further analysis. When aiming for results based on PCM, 2 M MgCl_2_ should be used. However, it must be recognized that lysis efficiency is highly dependent upon the anatomical organ and on any pathological changes than may have taken place. If pathological changes can be diagnosed, this is most commonly associated with changes in tissue composition or structure. The lysis buffer strategy should then be adapted accordingly. It is advisable to perform a process control for each experiment as suggested in [15], as recovery rates of target cells can be influenced by tissue changes.

Until now, prior to plating, an enrichment step has been necessary for detection of bacteria from tissues that do not readily permit target cell quantification [17]. The results of this study demonstrate that plating of bacteria and reliable quantification becomes possible implementation of a tissue preparation method that protects cultivable bacteria. With Matrix-Lysis, using the MgCl_2_ lysis buffer without detergent, the matrix size is reduced and targets are concentrated. We have shown that quantification of very low target cells, even a single cell, is reliable and reproducible using Matrix-Lysis for tissue preparation and the PCM method for quantification.

The constant mean recovery rate over the dilution series after PCM (96.1%; *R*^*2*^ *=* 0.99) revealed excellent stability of the method, independent of the target quantity. This offers new and promising strategies for quantitative plating approaches in analytical veterinary diagnostics. Matrix-Lysis, followed by quantification with qPCR, can produce the same mean recovery rates as published before using Matrix-Lysis for food matrices [25-27]. Processed kidney tissue was associated with lower recovery rates (15% ± 7.9%), even although kidney tissue resulted in a small pellet (<350 μg) after Matrix-Lysis and background DNA in the pellet was similar to that obtained from other organs (data not shown). This may suggest that the lower recovery rate is consequential to inadequate removal of inhibitors from the remaining pellet. Improved processing of kidney tissue will be examined further.

Spiking experiments with cerebrum over the whole dilution series resulted in a very high coefficient of determination (*R*^*2*^ *=* 0.99). For the lysis buffers based on 8 M urea + 1% SDS and 2 M MgCl_2_ + 1% Lutensol, constant recovery down to 349 BCE/g and 406 BCE/g, respectively, could be achieved with qPCR. As shown in this study, the newly developed Matrix-Lysis tissue preparation method is capable of recovering and concentrating single bacterial cells from as much as 5 g of tissue sample, efficiently and robustly.

Nevertheless, the discrepancy between the LOD of one cell with PCM compared to that of 10^2^ cells with qPCR has to be discussed. Firstly, most qPCR approaches use only a small fraction (commonly between 1/20 – 1/40; this study 1/20) of the sample eluate after DNA-extraction, which inevitably increases the overall detection limit [28]. Secondly, it is well known that common DNA purification procedures are not highly efficient at recovering target DNA at low concentrations from a high amount of background DNA; which may also lead to an increase in the LOD [28]. Finally, in this study only 1/2 of the B3 buffer lysate (part of the DNA extraction system used) was taken for DNA extraction when quantifying with qPCR. The reason for this was the remaining tissue DNA in the pellet. Although Matrix-Lysis reduced 5 log units of free DNA from the sample [26], the column of DNA extraction was overloaded with DNA from the remaining pellet after Matrix-Lysis. This leads to an underestimation of target cells. Processing half of this amount of the B3 buffer lysate (part of the DNA extraction system used) can easily circumvent this limitation, but it increases the detection limit.

Additional file 1: Figure S1 depicts the decrease in target copy detection with more than 70 μg of background DNA in the DNA extraction system. Background DNA content of the Matrix-Lysis pellets was approximately 200–400 μg.

If only qualitative results are required, novel approaches, such as molecular enrichment, can circumvent these limitations [28] and this is an important methodological consideration when using qPCR in the future. However, this was not applied in this study. If there is a goal for future detection limits as low as ~100 BCE/5 g, these results also highlight the urgent need for more efficient and robust DNA-extraction and concentration methods.

As previously indicated, DNA-extraction is an important bottleneck step for qPCR-based quantification and from the results of this study we suggest the application of internal cellular-based extraction controls, to control for inefficient extraction and purification [17]. Nevertheless, the detection limits achieved with Matrix-Lysis and qPCR are as good as the quantification limits associated with high-technology methods, such as fiber optic immunosensors coupled with immunomagnetic separation or antibody-based immunosensors [29,30]. To our knowledge, Matrix-Lysis is the only economical and easy-to-use tissue preparation method that can compete with these novel high-technology methods, considering the LOD and coefficients of determination in whole dilution series.

## Conclusions

In summary, it can be concluded that the Matrix-Lysis protocol from food science can be successfully adapted to clinical tissues. To our knowledge this is the first study presenting an easy-to-use and economical tissue preparation method for routine use and analytical veterinary diagnostics. The tissue preparation method permits quantification of pathogens from 5 g samples of organic matrix, which is 20–200 fold higher compared to prior sample preparation methods. Following Matrix-Lysis, the remaining pellet contains the total bacterial population of the sample. This pellet can then be further processed with DNA-based quantification or plating methods. It can be used for clinical research and diagnostic monitoring. For example, enumeration of lactobacilli and other Gram-positives can be useful for studying effects of dietary manipulations in pigs [31] or pathogen quantification can be used to support inoculation studies (e.g. vaccine testing). Hazard assessments and slaughterhouse practices will strongly profit from this reliable tissue preparation method and it is anticipated that this study will be a valuable source for further research on tissue preparation strategies.

## Methods

### Animal tissues

Cerebrum, spinal cord, lung, liver, ileum, colon, caecum, kidney and muscle tissues were obtained from slaughtered pig carcasses from a slaughterhouse in lower Austria and from tissue provided from the Clinic for Swine, University of Veterinary Medicine, Vienna. No animals were euthanized for the purposes of this research. Exact anatomic sampling sites are listed in Table 3. The tissues were cut into 5 g pieces and inserted into 50 ml polypropylene tubes for storage (Cornig, NY, USA). All tubes were cooled during transport and stored at −20°C.

**Table 3:** Animal tissues and precise anatomical sampling locations for all tissues used in this study

**Tissue**	**Sampling location**
Cerebrum	*Substantia grisea* and *Substantia alba*
Spinal cord	*Pars cervicalis*
Liver	*Lobus hepatis dexter lateralis*
Colon	*Colon ascendens*
Kidney	Border medulla - Cortex
Lung	Right lung: *Lobus medius, Lobus caudalis*
Ileum	Terminal ileum, sparing *Plica ileocaecalis*
Caecum	*Apex caeci*
Muscle	Cervical musculature

### Culture of Listeria monocytogenes EGDe

*L. monocytogenes* (1/2a, internal number 2964) was conserved at −80°C using MicroBank technology (Pro-Lab Diagnostics, Richmond Hill, Ontario, Canada) and constituted part of the collection of bacterial strains kept by the Institute of Milk Hygiene, University of Veterinary Medicine, Vienna. *L. monocytogenes* was incubated overnight (37°C) in tryptone soy broth (TSB) with 0.6% (w/v) yeast extract (Oxoid, Basingstoke, Hampshire, UK).

### Spiking of clinical tissues and experimental controls

For artificial contamination of tissues a 1 ml aliquot was taken from the overnight culture, placed into fresh TSB and directly incubated at 37°C for another three hours. Subsequently, a ten-fold dilution series was prepared using phosphate buffered saline (PBS) as diluent. The amount of inocula (100 μl) for spiking dilution series using cerebral tissue ranged from approximately 10^1^- 10^6^ BCE or respectively CFU. For the spiking trial of nine different tissues, a target concentration of approximately 10^5^ BCE/g was used. Ice-cold tissues were cut into small pieces (Ø <4 mm^2^) and spiked with pipetted droplets of culture of known concentration. Tissues were processed immediately. All spiking experiments and determination of the LOD were performed in triplicate. All organs used for artificial contamination experiments were tested to be negative for *L. monocytogenes* prior to inoculation using the Matrix-Lysis protocol and the real-time PCR assay described by Rossmanith et al. [32].

All spiking experiments were controlled by three methods: using the plate count method (PCM), microscopic enumeration and a qPCR step to check for possible discrepancies between the different enumeration techniques. Tryptone soy agar plates, supplemented with 0.6% (w/v) yeast extract (TSAYE; Oxoid), were used for the PCM. Dilution series of the cultures were plated and incubated at 37°C for 24 hours. Microscopic investigation was performed with 500 μl of an appropriate dilution of bacteria, following the protocol provided in the Live/Dead® BacLight kit (Molecular Probes, Willow Creek, OR, USA). Samples were analyzed using a Laborlux 8 fluorescence microscope (Leitz, Wetzlar, Germany) with a 470 nm filter and a 1,000-fold optical magnification. Two filters were analyzed per sample and fifteen visual fields were counted per filter. The following formula was used for calculation of the number of stained bacteria per milliliter: mean bacterial count in one field X (filtration area size/field size) X (1/dilution factor × 0.5 ml).

### Matrix-Lysis protocol and its adaptation to tissue matrices

The solubilizing ability of different buffer systems was examined for testing the applicability of Matrix-Lysis on clinical tissues. Matrix-Lysis buffers (sucrose + protease buffer, lysing buffer 8 M urea + 1% SDS, washing buffer) were prepared as previously described [26]. Matrix-Lysis buffers based on magnesium chloride comprised 2 M MgCl_2_, 50 mM Tricine and optionally 1% Lutensol AO-07 and a pH adjusted to 7.0 (P. Mester, personal communication). All reagents for the Matrix-Lysis protocol were purchased from Merck (Darmstadt, Germany), with the exception of SDS, Tricine (Sigma-Aldrich, Steinheim, Germany), Lutensol AO-07 (BASF, Arnheim, Netherlands) and Savinase (Novozymes, Krogshoejvev, Denmark).

In this study the Matrix-Lysis protocol was tested using the lysis buffer 8 M urea + 1% SDS, and both variants of MgCl_2_ lysis buffers, with and without 1% Lutensol. The Matrix-Lysis protocol previously published [26] was followed. After determining the resultant pellet size of different tissues after the Matrix-Lysis preparation step, the protocol was adapted for optimum solubilization of clinical tissue. The adapted protocol included five grams of clinical tissue that were cut into small pieces (Ø <4 mm^2^) and homogenized with the Stomacher 400 blender (Steward, London, UK) in 25 ml of sucrose-protease buffer for ten minutes. The homogenate was transferred to a sterile 50 ml polypropylene tube (Cornig, NY, USA) and incubated in a water bath at 37°C shaken at 250 rpm for 30 minutes. Samples were then centrifuged at 3,220 × g at room temperature for 30 minutes. The supernatant was decanted and the pellet resuspended in 30 ml lysis buffer. Samples were then incubated in a water bath at 37°C (lysis buffer based on MgCl_2_) or 42°C (lysis buffer based on urea) and shaken at 250 rpm. Subsequently, samples were re-homogenized for ten minutes. Resultant sample homogenates were then made up to 45 ml with lysis buffer and vortexed rigorously. Diluted homogenates were centrifuged at 3,220 × g for 30 minutes at room temperature. Supernatants were decanted and pellets resuspended in 40 ml of washing buffer. Samples were then incubated in a water bath for 30 minutes (shaken at 250 rpm), at the same temperature used during incubation in lysis buffer. Incubated samples were next centrifuged at 3,220 × g for 30 minutes at room temperature and the supernatant decanted. Pellets were resuspended in 1,500 μl PBS and transferred to a 2 ml plastic tube (Eppendorf, Hamburg, Germany). Pellets were washed twice using 1,500 μl PBS in centrifugation steps (5 minutes, 8,000 × g). The adapted protocol is shown in Figure 1. All buffers (protease buffer, lysis buffer and washing buffer) were pre-warmed before use and all experiments were performed in triplicate.

### Plating, DNA extraction and qPCR

For quantification of *L. monocytogenes* in clinical tissues after Matrix-Lysis, the remaining pellet was either plated onto selective agar plates for viable cell quantification or DNA extraction was performed for subsequent qPCR enumeration. The PCM was used for viable cell quantification. The remaining pellet from Matrix-Lysis was resuspended in PBS to achieve a total volume of one ml and rigorously vortexed until homogenized. 100 μl aliquots of a ten-fold dilution series were plated onto selective ALOA agar (Biolife, Milan, Italy). DNA extraction was performed with the NucleoSpin® tissue kit (Machery-Nagel, Düren, Germany) using the protocol for Gram-positive bacteria.

Since somatic cells remain in the Matrix-Lysis pellet and result in high background DNA levels and overload of the DNA extraction column, only 1/2 of the B3 buffer lysate was used for further DNA extraction. Following extraction, qPCR quantification of *L. monocytogenes* was performed by targeting a fragment of the *prfA* gene (274 bp) [32,33]. A DNA standard containing 1 ng/μl of *L. monocytogenes* DNA and a derivative 4 log_10_ dilution series were used as calibration standards for each run. The qPCR reaction on 25 μl samples was performed in a Mx3000p qCR termocycler (Stratagene; La Jolla, CA, USA) in duplicate using 5 μl of DNA template.

Supplementary Information accompanies the paper on the BMC Veterinary Research Journal website (http://biomedcentral.com).

## Competing interest

All authors disclose that no financial and/or personal relationships with other people or organizations exist that could inappropriately influence (bias) their work.

## Authors’ contributions

EM performed the experimental work, analysed the results and composed the manuscript. KP and PM participated with the spiking experiments and real-time PCRs. MW participated in the design of the study. PM and PR helped with design and coordination of this study and in editing the manuscript. All authors read and approved the final manuscript.

## Supplementary Material

Additional file 1: Figure S1 Influence of background DNA concentration on DNA extraction efficiency. 30–300 μg of salmon sperm-DNA was added to the DNA extraction system to determine changes in quantification efficiency in the presence of background DNA. Click here for file
