# Supplementary figures and images for: Quantification of Gram-positive bacteria: adaptation and evaluation of a preparation strategy using high amounts of clinical tissue
Source: BMC Vet Res. 2014 Mar 3;10:53. doi: 10.1186/1746-6148-10-53 (PMC4015715; doi:10.1186/1746-6148-10-53)

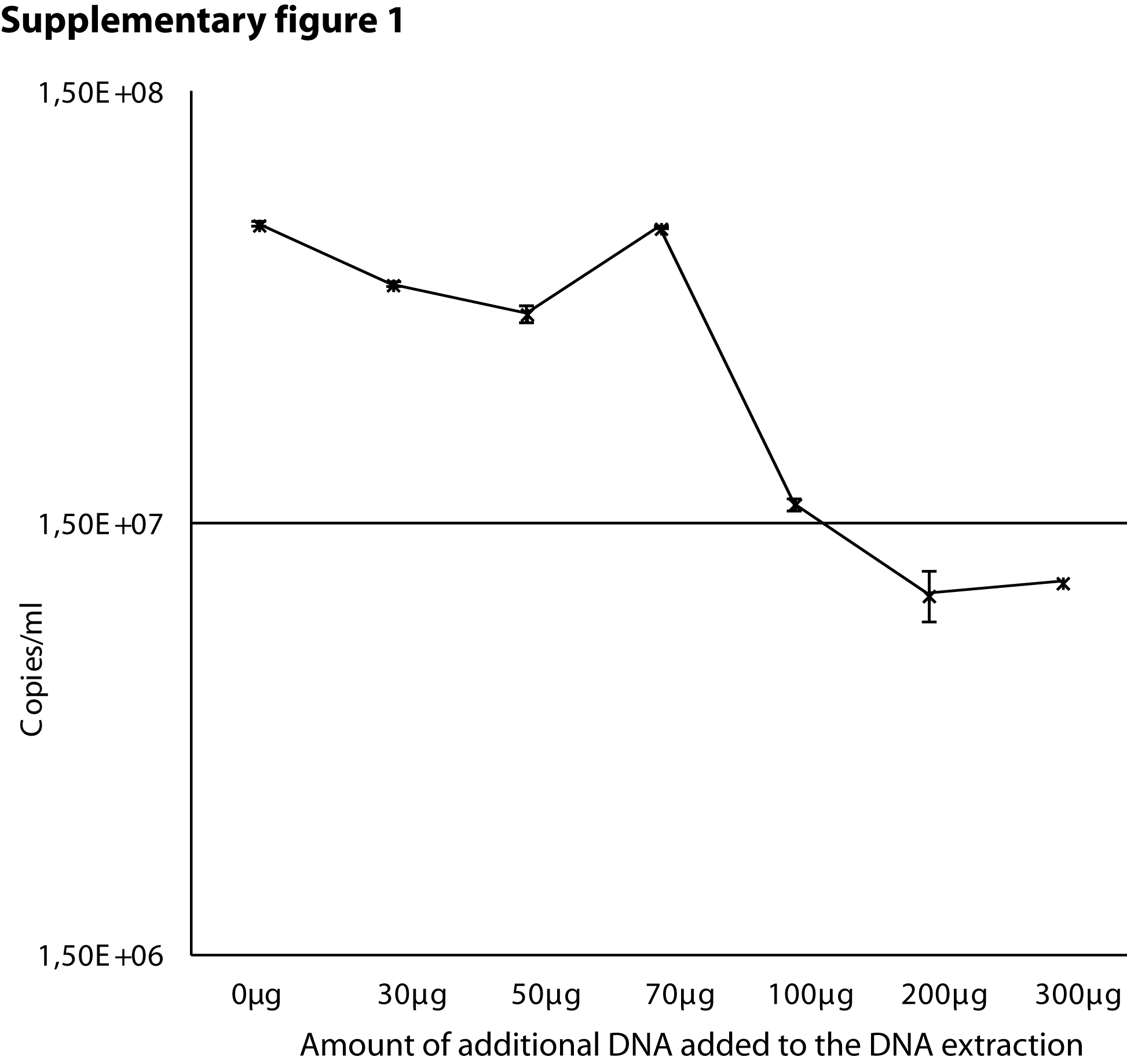

Supplement: Additional file 1: Figure S1 — Influence of background DNA concentration on DNA extraction efficiency. 30–300 μg of salmon sperm-DNA was added to the DNA extraction system to determine changes in quantification efficiency in the presence of background DNA. [file 1746-6148-10-53-S1.png]
